# Chronic Stress Is Associated with Pain Precipitation and Elevation in DeltaFosb Expression

**DOI:** 10.3389/fphar.2016.00138

**Published:** 2016-05-30

**Authors:** Hang Wang, Xinrong Tao, Si-Ting Huang, Liang Wu, Hui-Li Tang, Ying Song, Gongliang Zhang, Yong-Mei Zhang

**Affiliations:** ^1^Jiangsu Province Key Laboratory of Anesthesiology, College of Anesthesiology, Xuzhou Medical UniversityXuzhou, China; ^2^College of Medicine, Anhui University of Science and TechnologyHuainan, China; ^3^School of Basic Medical Sciences, Anhui Medical UniversityHefei, China

**Keywords:** Deltafosb, ΔFosB, medial prefrontal cortex, pain, western blot

## Abstract

A number of acute or repeated stimuli can induce expression of DeltaFosB (ΔFosB), a transcription factor derived from the fosB gene (an osteosarcoma viral oncogene) via alternative splicing. ΔFosB protein is currently viewed as a ‘molecular switch’ to repeated stimuli that gradually converts acute responses into relatively stable adaptations underlying long-term neural and behavioral plasticity. ΔFosB has received extensive attention in drug addition, depression, and stress adaptation, but changes in ΔFosB protein expression during pain is not fully understood. In this study we explored ΔFosB expression in the medial prefrontal cortex (mPFC) of rats experiencing chronic or acute stress-induced pain. Our data reveal that chronic pain induced by neonatal colorectal distension, chronic constriction injury (CCI) of the sciatic nerve, or maternal separation was associated with an increase in ΔfosB protein expression in mPFC, but acute application of acetic acid or zymosan did not alter the ΔFosB protein expression. ΔFosB expression in the rat visual cortex, a non pain-related brain region, did not change in response to (CCI) of the sciatic nerve and acetic acid treatment. In conclusion, our results indicate that ΔFosB protein expression is significantly elevated in rats that have experienced chronic pain and stress, but not acute pain. The ΔFosB protein may serve as an important transcription factor for chronic stress-induced pain. Further research is needed to improve the understanding of both the upstream signaling leading to ΔFosB protein expression as well as the regulation of ΔFosB gene expression in cortical neurons.

## Introduction

Pain is associated with an alteration in gene expression and neuronal plasticity (Baliki et al., 2014) that can result in persistent structural and functional modifications (Woolf and Costigan, 1999; Ji et al., 2014). Transcription factors bridge the pathological events in the central nervous system (CNS) with alterations in gene expression during the pathogenesis of pain (Woolf and Costigan, 1999; Zhang et al., 2015). Transcription factors belonging to the Fos family may be important candidates for pain modulation (Luis-Delgado et al., 2006). Encoded by *c-fos*, *fosB*, *fra-1*, and *fra-2* genes (Herdegen and Leah, 1998), Fos family proteins are induced rapidly and transiently in specific brain regions to regulate downstream gene expression in response to environmental factors (Sheng and Greenberg, 1990; Perrotti et al., 2004). These Fos family proteins form heterodimers with Jun family proteins (c-Jun, JunB, or JunD) to form functioning activator protein-1 (AP1) transcription factors that bind to AP1 consensus sites present in the promoters of certain genes to regulate their transcription. The DeltaFosB (ΔFosB) protein is a truncated splice variant of *fosB*, missing 101 amino acids at the C-terminal of the FosB protein (Nakabeppu and Nathans, 1991). Recently the ΔFosB protein has received extensive attention in drug addition, depression and stress adaptation (McClung and Nestler, 2003; Perrotti et al., 2004).

The ΔFosB protein is encoded by the fosB gene and shares homology with other Fos family proteins (Morgan and Curran, 1995). ΔFosB has the same dimerization and DNA-binding activities of FosB. Upon stress stimulus, the post-translational modifications of ΔFosB protein lead to different protein isoforms with half-lives between 10 h and 8 days (McClung et al., 2004). Therefore, ΔFosB protein is assumed to function as a “molecular switch” that mediates persistent adaptations in the brain’s response to repetitive or long-lasting stimuli (Nestler et al., 1999, 2001). In the CNS, ΔFosB has been examined in the context of electroconvulsive treatment and epilepsy (Morris et al., 2000), addiction (McClung and Nestler, 2003; Zhang et al., 2014), compulsive running (Werme et al., 2002), dyskinesia (Andersson et al., 2003), and stress (Perrotti et al., 2004). For instance, Zhang reported that ΔFosB overexpression decreases cocaine self-administration, enhances extinction of cocaine seeking, and decreases cocaine-induced reinstatement of intravenous cocaine self-administration (Zhang et al., 2014). However, limited evidence suggests an involvement of ΔFosB protein in acute or chronic stress-induced pain precipitation. Mice overexpressing the ΔFosB protein in the nucleus accumbens and the dorsal striatum displayed reduced analgesic responses to acute morphine administration as well as faster development of morphine tolerance (Zachariou et al., 2006). Carrageenan-induced inflammation increases ΔFosB protein expression in the spinal cord (Luis-Delgado et al., 2006). These reports suggest that ΔFosB may be an important molecular modulator participating in the pathogenesis of pain. To date, the direct evidence of changes in ΔFosB protein expression in various pain models has not been reported.

Pain is associated with depression and cognitive decline (Metz et al., 2009; Zhang et al., 2015), suggesting an involvement of cortical areas associated with cognitive functions. The medial prefrontal cortex (mPFC) plays a critical role in pain-related depression and anxiety, which are frequent co-morbidities of chronic pain (Guida et al., 2015). Unilateral chronic constriction injury (CCI) to the infraorbital nerve induces a strong, bilateral upregulation of extracellular signal regulated kinases 1/2 (pERK-1/2) in the ventral mPFC in rats (Devoize et al., 2011). Stress induces ΔFosB protein expression in mPFC, and that overexpression of ΔFosB in pre-limbic mPFC enhances stress susceptibility in mice (Vialou et al., 2014). mPFC may be a key brain region through which the ΔFosB protein modulates the establishment and maintenance of pain.

In the present study, we examined ΔFosB protein expression in mPFC in rodents that experienced acute and chronic stress-induced pain. The visual cortex, a non pain-related brain region, was used as a control. Our data reveal that chronic stress-induced pain was associated with an increase in the expression of mPFC ΔFosB protein, but ΔFosB expression did not change in rats that experienced acute stress-induced pain. These results illuminate a new molecular mechanism of pain, and may pave a new avenue for development of therapeutics against pain.

## Materials and Methods

### Animals

Male Sprague-Dawley rats weighing 220 to 250 g and C57B mice weighing 21 to 24 g were obtained from the Experimental Animal Center of Xuzhou Medical University (Xuzhou, China) and housed in groups of four per standard polycarbonate cage and kept on a standard 12 h light/dark cycle (lights on at 07:00 a.m.), constant temperature and humidity (22°C and 50%, respectively) with food provided *ad libitum*. All procedures were conducted in accordance with the guidelines described in the National Institutes of Health’s *Guide for the Care and Use of Laboratory Animals* (NIH Publication No. 8023, revised 1978) and the International Association for the Study of Pain, and approved by the Institutional Animal Care and Use Committee at Xuzhou Medical College.

### Development of Pain Models

#### Development of Visceral Hypersensitivity Rat Model with Colorectal Distensions

Visceral hypersensitivity was induced by adult colorectal distension (*CRD*) in rats that experience neonatal *CRD*s as described previously (Chen et al., 2015; Zhang et al., 2015). In brief, neonatal *CRDs* were induced on postnatal days 8, 10, and 12 using an angioplasty balloon (length, 20.0 mm; diameter, 3.0 mm) inserted into the upper rectum and descending colon (the section of the large intestine that travels back down toward the rectum). The balloon was distended with 0.3 ml water at a pressure of 60 mm Hg for 1 min before deflation and withdrawal. The distention was repeated twice with a 30-min break. *CRD* for adult rats was established 8 weeks later in which an 80 mm Hg (1 min) distention was given twice with a 5 min interval. The extent of visceral hypersensitivity was assessed with abdominal withdrawal reflex (AWR) scores, pain threshold, and electromyography activities of oblique muscles as described previously (Zhang et al., 2015).

#### Chronic Constriction Injury (CCI) Neuropathic Pain Model

The chronic neuropathic pain model was generated using a chronic sciatic nerve compression injury method (Bennett and Xie, 1988). Rats were anaesthetized with intraperitoneal injection of 10% chloral hydrate at 400 mg/kg. After anaesthetization and disinfection, the sciatic nerve trunk was isolated and ligated for a total of four ligations at an interval of 1 mm. The sciatic nerve was exposed without ligation treatment in the control group. Paw withdrawal latency (PWL) was used to evaluate the pain level.

#### Maternal Separation Model

The protocol of maternal separation was conducted as previously described in detail (Wang et al., 2015). The male and female rats were mated to produce litters. After birth, the pups were randomly divided into two groups: the maternal separation group and the non-maternal separation group. Neonatal mice were separated from mothers for 4 h (10:00 a.m.–2:00 p.m.) per day ranging from postnatal day 1 to day 15, and maintained on a water-heating pad (29 ± 1°C) separately from their littermates. The pups in non-maternal separation group remained in their home cages with their mothers and littermates during the 4 h separation.

#### Acetic Acid-Elicited Acute Visceral Pain

Intraperitoneal acetic acid-induced abdominal contraction was used to establish the acute pain model (Martinez et al., 1999). In brief, mice were placed individually in a standard polycarbonate cage, and allowed to habituate to the environment for 30 min. Acetic acid (0.6% in distilled water) was injected intraperitoneally in a volume of 10 ml/kg. Immediately after the acetic acid or vehicle injection, pain responses were scored by counting the number of abdominal contractions (writhing test) in 15 min intervals for 1 h. An abdominal contraction was defined as a lengthwise stretching of the torso with a concomitant arching of the back.

#### Zymosan-Induced Paw Inflammatory Pain

Intraplantar injections of zymosan (1.25 mg/100 μl) were utilized to induce paw inflammation. Sham group received an equal volume of saline. The thermal latencies of the rats were measured pre- and 0.5, 1, 2, 4, 8, and 24 h post-injection.

### Immunofluorescence Labeling

Rats were deeply anesthetized with 10% chloral hydrate (250 mg/kg) and perfused transcardially with 300 ml 0.9% saline, followed by 4% paraformaldehyde. The entire brain was quickly removed and further fixed in 4% paraformaldehyde for 48 h at 4°C before being equilibrated in a 30% sucrose solution at 4°C for 2 days. Double immunofluorescence labeling was used to identify ΔFosB protein distribution in particular cell types. The mPFC region was cut in 30-μm thick slices with a cryostat (Leica CM1800; Heidelberg, Germany). Selected sections were washed with PBS for 5 min three times, and then incubated with 10% donkey serum in PBS containing 0.3% Triton-X-100 for 2 h at room temperature before incubation with rabbit anti-delta Fosb antibody (1:500, #9890, Cell Signaling Technology), anti-neuronal nuclei (NeuN) antibody (1:200; MAB377X, Millipore), anti-glial fibrillary acidic protein (GFAP; 1:200; #3670, Cell Signaling Technology), or anti-ionized calcium-binding adaptor molecule 1 (Iba-1; 1:200; ab5076, Abcam) at 4°C for 24 h. Alexa 488 donkey anti-rabbit IgG (1:200), Alexa 594 donkey anti-mouse IgG (1:200), or Alexa 594 donkey anti-goat IgG (1:200) were added to the corresponding sections and incubated for 2 h at room temperature. Tissue sections were mounted with 50% glycerol mounting medium. Layer V of the prelimbic cortex on the left hemisphere in each mouse was visualized with a confocal laser microscope (FV1000; Olympus, Tokyo, Japan). Tissue images were processed using Image Pro-Plus 5.0 software (Media Cybernetics, Silver Spring, MD).

### Western Blotting Analysis

The bilateral mPFC and visual cortex were collected from fresh brain and lysed in RIPA lysis buffer containing 50 mM Tris (pH 7.4), 150 mM NaCl, 1% Triton X-100, 1% sodium deoxycholate, 0.1% SDS, 1 mM sodium orthovanadate, and 1 mM Phenylmethanesulfonyl fluoride (PMSF). Homogenates were keep on ice for another 30 min before being centrifuged at 8,000 *g* for 5 min at 4°C; the supernatant containing the cytoplasmic components was saved at -80°C before analysis. Equal amounts of protein (80 μg) were separated by SDS-PAGE, transferred electrophoretically to nitrocellulose membrane, and incubated with rabbit polyclonal anti-ΔFosB overnight at 4°C. The membrane was washed thoroughly and incubated with AP-conjugated secondary antibody (1:1000) for 2 h at room temperature. Protein bands were visualized using the BCIP/NBT Alkaline Phosphatase Color Development Kit and quantified using Image J software (National Institutes of Health).

### Statistical Analysis

Data were expressed as mean ± SEM. The time-course of the number of abdominal contraction was analyzed by a two-way repeated measures ANOVA. Student’s *t*-test, one-way ANOVA, and two-way ANOVA were also used. If significance was found, *post hoc* Bonferroni multiple comparison was used. All statistical tests were conducted using SPSS 19.0 software package (IBM, Armonk, NY, USA) with *p* < 0.05 considered statistically significant.

## Results

### Neonatal CRD Exposure is Associated with an Increase in Adult ΔFosB Protein Expression in mPFC

**FIGURE 1:**
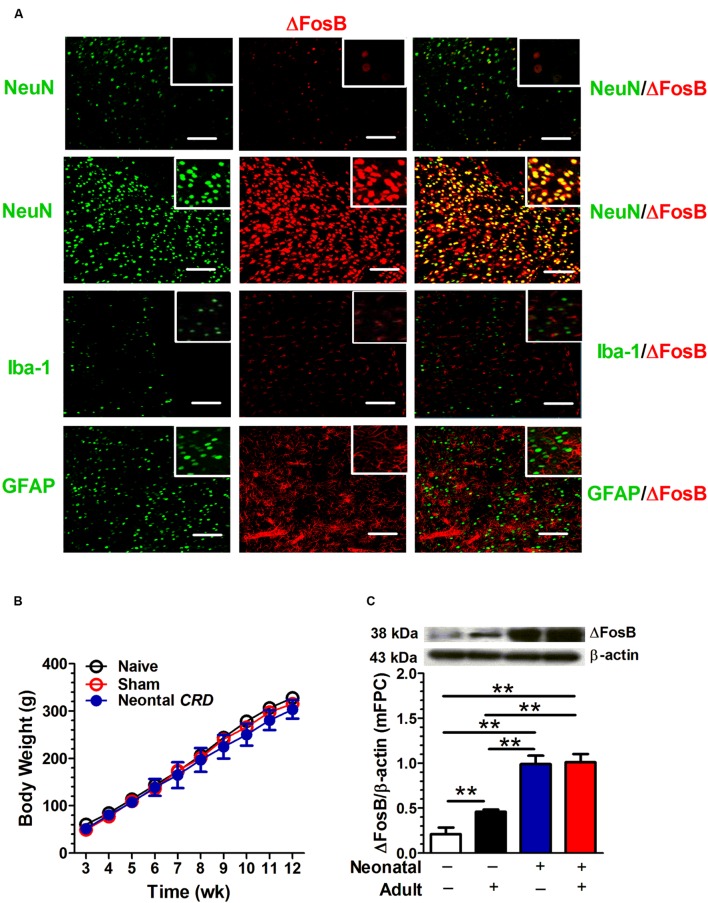
**Neonatal colorectal distension (CRD) exposure is associated with an increase in adult ΔFosB expression in mFPC.** ΔFosB protein labeling was compared with neuronal marker NeuN, microglial marker Iba-1 and astrocyte marker GFAP. ΔFosB was visualized in pyramidal neurons marked by NeuN staining. Row one came from drug-naïve rats, and rows 2 to 4 was from rats that experienced neonatal and adult *CRD*s. Scale bar = 100 μm **(A)**. Mice received neonatal and/or adult colorectal distension. There was no difference in body weight. (**B**; *n* = 8 per group). A two-way ANOVA on ΔFosB expression revealed a significant main effect of neonatal *CRD*s (*p* < 0.01) and a significant main effect of adult *CRD*s (*p* < 0.05). Rats that experienced neonatal or a combination of neonatal and adult *CRD*s presented a significant increase in ΔFosB expression compared with rats without *CRD*s or adult *CRD* only **(C)**. Data were expressed as mean ± SEM, *n* = 3 in each group. **p < 0.01.

The ΔFosB protein labeling was compared with neuronal marker NeuN, microglial marker Iba-1 and astrocyte marker GFAP. The top row in **Figure 1A** shows the ΔFosB and NeuN staining in prelimbic area of drug-naïve rats. Rows 2–4 in **Figure 1A** illustrate protein staining in rats that had experienced neonatal and adult *CRD*s. As compared to drug-naïve rats, the rats experienced *CRD*s presented a significant increase in NeuN and ΔFosB immunoreactivity. ΔFosB was visualized in pyramidal neurons marked by NeuN staining (**Figure 1A**). A two-way ANOVA on body weight revealed a significant main effect of neonatal *CRD*s [*F*(1,8) = 218.56, *p* < 0.01], a significant main effect of adult *CRD* [*F*(1,8) = 8.80, *p* < 0.05], but not the neonatal × adult *CRD*s interaction [*F*(1,8) = 6.39, *p* = 0.035] (*n* = 8 in each group; **Figure 1B**). As compared to naïve rats, there was a significant increase in ΔFosB protein expression in rats that had experienced neonatal *CRD*s [*t*(4) = 11.46, *p* < 0.01], adult *CRDs* [*t*(4) = 4.98, *p* < 0.01] or a combination of neonatal and adult *CRD*s [*t*(4) = 11.78, *p* < 0.01]. Adult *CRD* rats presented a significantly lower expression of ΔFosB protein levels as compared with rats that had experienced neonatal *CRD*s [*t*(4) = 8.99, *p* < 0.01] or a combination of neonatal and adult *CRD*s [*t*(4) = 0.9.35, *p* < 0.01]. There was no difference between rats that had experienced neonatal *CRD*s and those with a combination of neonatal + adult *CRDs* [*t*(4) = 0.26, *p* = 0.81] (**Figure 1C**).

### Chronic Constriction Injury Is Associated with an Increase in Adult ΔFosB Protein Expression in mPFC

**FIGURE 2:**
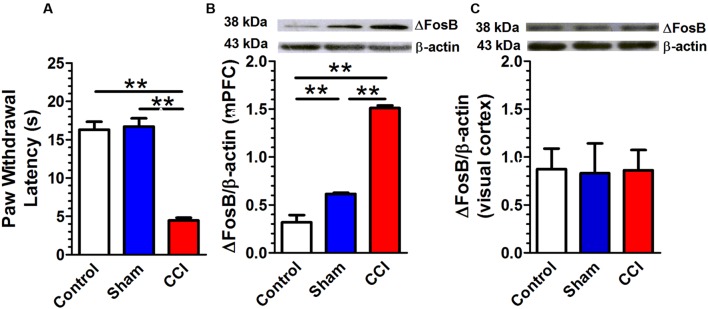
**Chronic constriction injury (CCI) is associated with an increase in adult ΔFosB expression in mPFC.** Rats that received CCI of the sciatic nerve presented a significant decrease in paw withdrawal latency (PWL; **A**; *n* = 17 per group) and a significant increase in ΔFosB expression. (**B**; *n* = 4 per group) compared with control or sham rats. There was no change in ΔFosB expression in the visual cortex between control, sham and CCI groups. (**C**; *n* = 3 per group). Data were expressed as mean ± SEM, ^∗∗^*p* < 0.01.

The CCI in rats altered the PWL score (**Figure 2A**). A one-way ANOVA showed a significant difference [*F*(2,48) = 61.51, *p* < 0.01]; *Post hoc* Bonferroni’s multiple comparisons showed that CCI rats (*n* = 17) presented a significant decrease in PWL as compared to control (*n* = 17; *p* < 0.01) or sham rats (*n* = 17; *p* < 0.01). There was no difference between control and sham rats. The CCI in rats altered the ΔFosB protein expression (**Figure 2B**). A one-way ANOVA showed a significant difference [*F*(2,9) = 174.7, *p* < 0.01]; *Post hoc* Bonferroni’s multiple comparisons CCI rats (*n* = 4) presented a significant increase in ΔFosB expression as compared to control (*n* = 4; *p* < 0.01) and sham rats (*n* = 4; *p* < 0.01). Sham rats presented a significant increase in ΔFosB protein expression as compared to control rats (*p* < 0.01). On the other hand, ΔFosB protein expression in the visual cortex was not statistically different between control, sham and CCI rats [*F*(2,6) = 0.02, *p* = 0.98; *n* = 3 per group; **Figure 2C**].

### Maternal Separation Is Associated with an Increase in Adult ΔFosB Expression in mPFC

**FIGURE 3:**
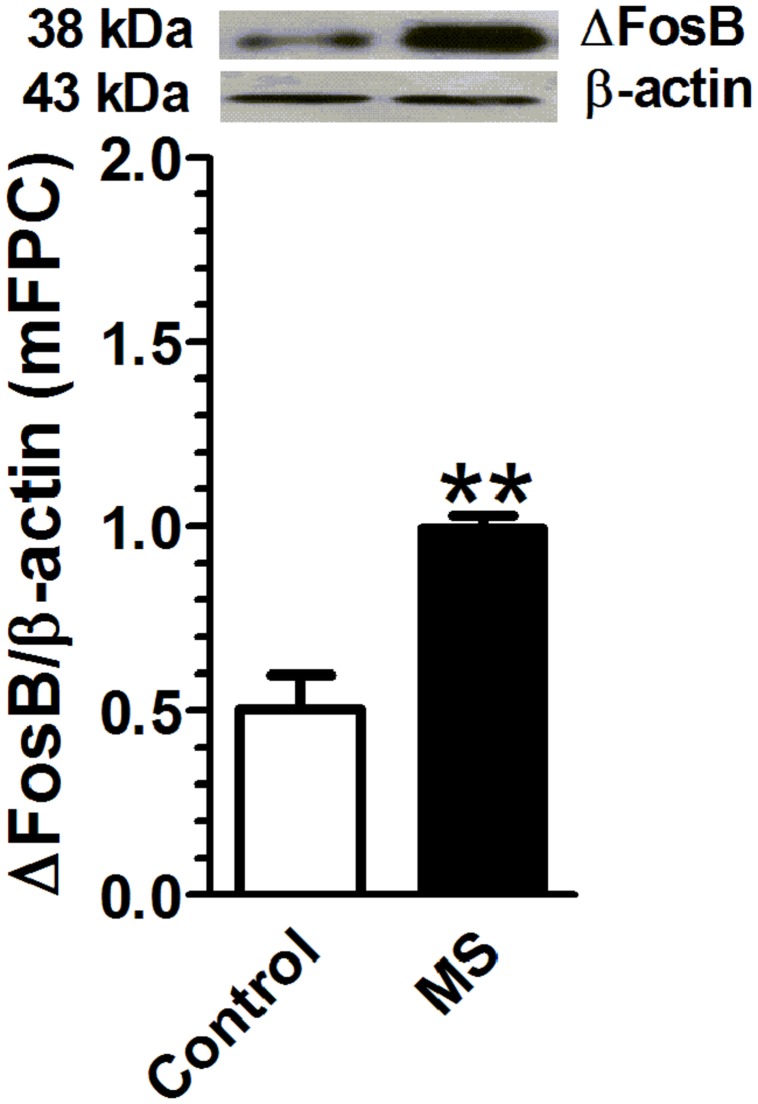
**Maternal separation is associated with an increase in adult ΔFosB expression in mPFC.** Rats exposed to maternal separation exhibited a significant increase in ΔFosB expression in mPFC compared with that in the control group (*p* < 0.01, *n* = 3). Data were expressed as mean ± SEM, ^∗∗^*p* < 0.01 vs. control.

Rats that underwent maternal separation presented a significant increase in ΔFosB protein expression in mPFC compared with that in the control group [*t*(4) = 4.95, *p* < 0.01, *n* = 3 per group; **Figure 3**].

### Acetic Acid-Induced Acute Visceral Pain Does Not Affect ΔFosB Expression in mPFC

**FIGURE 4:**
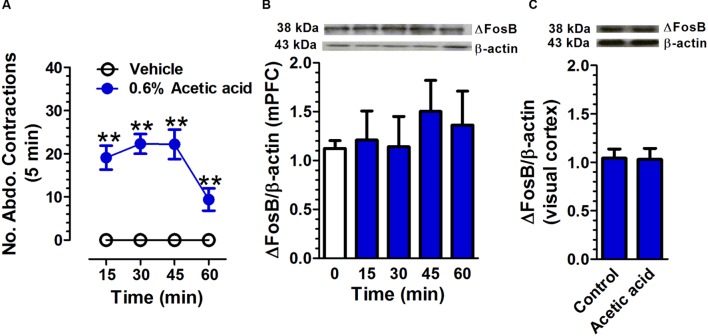
**Acetic acid-induced acute pain does not affect ΔFosB expression in mPFC.** Intraperitoneal injection of 0.6% acetic acid increased the number of abdominal contractions compared with that in control group. (**A**; *n* = 10 per group). The ΔFosB expression in mPFC was not significantly altered 15, 30, 45, and 60 min after acetic acid application. (**B**; *n* = 3 per group). There was no change in ΔFosB expression in the visual cortex between control and acetic acid-treated groups. (**C**; *n* = 3 per group; brain tissues were collected at 45 min after acetic acid treatment). Data were expressed as mean ± SEM, ^∗∗^*p* < 0.01 vs. vehicle.

Intraperitoneal injection of 0.6% acetic acid increased the number of abdominal contractions compared with that in the control group (**Figure 4A**). A two-way repeated measures ANOVA revealed a significant main effect of treatment [*F*(1,18) = 599.51, *p* < 0.01], a significant main effect of time [*F*(3,54) = 123.39, *p* < 0.01], and a significant time × treatment interaction [*F*(3,54) = 123.39, *p* < 0.01, *n* = 10 per group]. There was no significant difference in ΔFosB expression in mPFC between acetic acid group and control group [*F*(4,19) = 0.32, *p* = 0.86, *n* = 3; **Figure 4B**]. The ΔFosB expression in the visual cortex was not found to be statistically different between vehicle and acetic acid-treated rats [*t*(4) = 0.14, *p* = 0.89; *n* = 3; **Figure 4C**].

### Zymosan-Induced Paw Inflammatory Pain Does Not Affect ΔFosB Expression in mPFC

**FIGURE 5:**
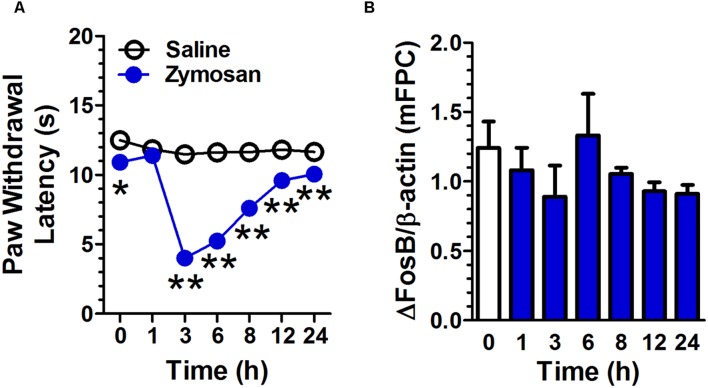
**Zymosan-induced paw inflammatory pain does not affect ΔFosB expression in mPFC.** Intrapaw injection of zymosan decreased the latency of paw withdrawal compared to that in the control group. (**A**; *n* = 6 per group). The ΔFosB expression in mPFC was not significantly altered 1, 3, 6, 8, 12, and 24 h after zymosan application. (**B**; *n* = 3 per group). Data were expressed as mean ± SEM, ^∗∗^*p* < 0.01 vs. saline.

Intrapaw injection of zymosan decreased the latency of paw withdrawal compared with that in control group (**Figure 5A**). A two-way repeated measures ANOVA revealed a significant main effect of treatment [*F*(1,10) = 157.97, *p* < 0.01], a significant main effect of time [*F*(6,60) = 37.60, *p* < 0.01], and significant time × treatment interaction [*F*(6,60) = 27.84, *p* < 0.01, *n* = 6 per group]. There was no significant difference in ΔFosB expression in mPFC between zymosan-group and control group [*F*(6,14) = 0.97, *p* = 0.48, *n* = 3; **Figure 5B**].

## Discussion

In this study, we examined ΔFosB protein expression in animals that experienced acute or chronic stress-induced pain. Our data reveal an increase in ΔFosB expression in animals with chronic pain induced by CRD, CCI, and maternal separation, but not in acetic acid-induced acute visceral pain and zymosan-induced acute inflammatory pain. ΔFosB protein expression in the visual cortex did not change as a result of CCI or acetic acid-induced pain. These results suggest that ΔFosB may be an important transcriptional factor that modulates neuronal adaptation specifically to chronic stress-induced pain. Elucidation of the molecular mechanisms that are responsible for the specific ΔFosB response to chronic pain provides new opportunities for therapeutic approaches to prevent acute pain translated into chronic pain.

Our data indicate that ΔFosB protein expression is increased in rats that experienced neonatal CRDs. Visceral hypersensitivity is a major contributor to irritable bowel syndrome and other disorders with chronic visceral pain (Chey et al., 2015). The pathogenesis of visceral hypersensitivity remains speculative due to undetectable structural abnormalities in the peripheral organs (Chen et al., 2015). In the present study, visceral hypersensitivity was developed by neonatal and adult CRDs in rats. We did not find any identifiable behavioral abnormalities in adult rats that experienced neonatal CRDs (Chen et al., 2015; Zhang et al., 2015), but adult re-exposure to CRD precipitated behavioral abnormalities and visceral pain hypersensitivity only in rats that experienced neonatal CRDs (Zhang et al., 2015). These results suggest that neonatal CRDs alter the neuronal traits, in spite of the body maintaining a normal phenotype through adaptation. Such a quiescent event can be prompted by a subthreshold stressor imposed late in life, and induces changes in genotype and phenotype, such as fear reaction, cognition, dysthymia, novelty-seeking, and pain perception (Charmandari et al., 2003). Our data indicate that the neonatal CRD is associated with a significant increase in ΔFosB protein expression in mPFC.

Chronic neuropathic pain is a prevalent and debilitating condition, affecting 7–18% of the population (Bouhassira et al., 2008; Toth et al., 2009). Clinical manifestation includes spontaneous pain, dysaesthesia, paraesthesia, allodynia, and hyperalgesia. The sensory symptoms are co-morbid with mental impairments, such as insomnia and depression. CCI of the sciatic nerve is a chronic neuropathic pain resulting from damage to the peripheral nervous system. The CCI model is a popular tool to study mechanisms for chronic neuropathic pain and to identify experimental compounds with analgesic properties that are palliative for chronic neuropathic pain. Unilateral CCI to the infraorbital nerve induces a strong, bilateral upregulation of pERK-1/2 in the ventral mPFC of rats (Devoize et al., 2011). Our data reveal an increase in the expression of ΔFosB in mPFC in rats that experienced CCI. Interestingly, CCI did not alter ΔFosB expression in the visual cortex, a non pain-related brain region.

Early life adversity, such as postnatal maternal separation, plays a central role in the development of psychopathologies during individual ontogeny. The adverse early life event is a risk factor for the development of psychiatric diseases in adult. In rats, maternal separation, which deprives pups of their mothers, has often been used as a model for early life adversity (Hall, 1998). Maternal separation has been demonstrated to induce behavioral and cognitive abnormalities, such as increased depressive and anxiety-like behaviors (Rentesi et al., 2010) and prepulse inhibition and learning deficits (Garner et al., 2007). Maternal separation has also been shown to decrease new born cells in the hippocampus and the granular cell in the dentate gyrus of juvenile and adult rats (Hulshof et al., 2011), and decrease the expression of brain-derived neurotrophic factor (BDNF) in the mPFC in young adult rats (Wang et al., 2015). These findings suggest that maternal separation can affect the neuroplasticity of rats. In line with these results, our data revealed an increase in the expression of ΔFosB in mPFC in rats that experienced maternal separation.

Local or intraperitoneal injection of zymosan, a polysaccharide component of the cell wall from Saccharomyces cerevisiae, or acetic acid, represents a self-resolving model of acute inflammation and pain, which has been widely used for the quantification of particular cell types and inflammation-related soluble factors (Cash et al., 2009). Intraplantar administration of zymosan in the rat hindpaw produces a reliable and quantifiable thermal and mechanical hyperalgesia accompanied by oedema that closely mimics the symptoms of inflammation in humans (Meller and Gebhart, 1997). Our data indicate that the expression of ΔFosB in mPFC is not altered in rats with acetic acid- or zymosan- induced acute pain. Since ΔFosB expression is altered only in chronic or persistent exposure to chronic stimuli, it is not surprising that we did not observe the ΔFosB change in either the acetic acid- or the zymosan-induced acute pain models.

Early life stress, such as neonatal CRD and maternal separation, is a potential contributor to visceral hypersensitivity and pain (Al-Chaer et al., 2000; Zhou et al., 2010). The developing brain undergoes rapid growth and is characterized by high turnover of neuronal wiring. Early life stress affects the developmental trajectory of the neurocircuitry in the CNS and alters vulnerability to subsequent stress during adulthood (Wouters et al., 2012). Early life stress may produce stable changes in neuronal plasticity, function, and communication, and increase susceptibility to developing the enhanced pain-like behavioral profiles and later-life psychopathology (Van den Bergh et al., 2008; Green et al., 2011; Amath et al., 2012). The ΔFosB protein is elevated in chronic, but not acute stress-induced pain models. It is posited that ΔFosB functions as a molecular switch that mediates persistent adaptations in the brain response to chronic pain (Luis-Delgado et al., 2006).

The ΔFosB protein is a unique transcription factor that plays an essential role in long-term adaptive changes in the brain associated with diverse conditions, such as drug addiction, Parkinson’s disease, depression, and antidepressant treatment. In CNS, ΔFosB expression is regulated in a regional- and cell-type-specific manner by many types of chronic perturbations (Nestler et al., 2001; Werme et al., 2002). Once induced, it persists for a long period of time due to its unusual stability. The transcriptional effects of ΔFosB are complex, because the protein can function as both a transcriptional activator and repressor. Progress has been made in identifying specific target genes for ΔFosB and in relating some of these genes to the cellular and behavioral actions of the ΔfosB protein. Future studies will help us to better understand the biochemical basis of the ΔfosB protein’s unique stability, as well as the precise molecular pathways through which this transcription factor produces its complex effects on neuronal plasticity and complex behavior (McClung et al., 2004).

The upstream and downstream signaling pathways of ΔFosB remain largely unknown. Different stimuli can invoke different molecular mechanisms to induce ΔfosB protein levels. Inflammation is a major contributor to pain. This inflammatory neuroplasticity is the consequence of a combination of activity-dependent changes in the neurons and specific signal molecules initiating particular signal-transduction pathways and altering gene expression (Woolf and Costigan, 1999). We recently demonstrated that activation of microglial toll-like receptor 4 (TLR4)/ myeloid differentiation factor 88 (MyD88)/ nuclear factor κB (NF-κB) signaling, as well as proinflammatory cytokines tumor necrosis factor-α (TNF-α) and interleukin-1β (IL-1β) facilitated the development of visceral hypersensitivity and pain (Chen et al., 2015; Zhang et al., 2015). The casual relation between inflammatory modulators and the ΔFosB protein needs further exploration.

The downstream of ΔFosB remains elusive. ΔFosB dimerizes predominantly with JunD to form a heterodimerized AP1 complex (Hiroi et al., 1998). Recent in vitro evidence has indicated that ΔFosB can also form homodimers (Jorissen et al., 2007). ΔFosB acts as either a transcriptional repressor or activator (McClung and Nestler, 2003). A plethora of factors participate in hyperalgesia and allodynia following peripheral tissue inflammation, including cyclin-dependent kinase 5 (CDK5; Chen et al., 2000), cholecystokinin (CCK)-B receptor, (Covington et al., 2010) NMDA receptor subunit 1 (NR1; Hiroi et al., 1998) and AMPA ionotropic glutamate receptor subunit 2 (GluR2; Kelz et al., 1999; Rygh et al., 2001). Such data from the literature suggest that the above target genes could be candidates of interest for future studies to understand the action of the ΔFosB protein in the CNS.

The chronic conditions tested here do not allow separation of pain processing in the mPFC due to stress, depression or anxiety. ΔFosB is a transcriptional regulator of stress (Nestler, 2015). Stress, e.g., pain, physical abuse, neonatal maternal separation, or exposure to an immune challenge can induce genetic, physiological and behavioral changes (Mayer and Collins, 2002). The ΔFosB protein accumulates within the same brain regions after repeated stress exposure, whereas other Fos family members show desensitization [i.e., reduced induction compared with initial drug exposures (Perrotti et al., 2004; Lehmann and Herkenham, 2011)]. Such accumulation of ΔFosB has been observed due to several forms of active stress, such as chronic restraint stress, chronic unpredictable stress, and chronic social defeat stress, whereas chronic social isolation decreases ΔFosB levels in nucleus accumbens (Vialou et al., 2010). Stress induces ΔFosB expression in mPFC, and that overexpression of ΔFosB in pre-limbic mPFC enhances stress susceptibility in mice (Vialou et al., 2014). The mPFC shows lower basal levels of ΔFosB immunoreactivity, but almost a threefold induction after chronic restraint stress in rats (Perrotti et al., 2004). We found rats that experienced neonatal CRD and maternal separation showed an elevation in the ΔfosB protein levels. Maladaptation of the stress system may impair neuronal development, and account for a number of endocrine, metabolic, autoimmune, and psychiatric disorders (Green et al., 2011; Amath et al., 2012). Antidepressants can reverse this social withdrawal syndrome by boosting ΔFosB. Moreover, ΔFosB is conspicuously depleted in brains of people who suffered from depression. Thus, induction of ΔFosB is a positive adaptation for coping with stress. Under most circumstances, stress, depression, anxiety and pain share common contributors and neurobiological inhibitors. In this study, we reported that chronic pain rat models presented an elevation of ΔFosB protein level in mFPC. Interestingly, the ΔFosB expression in the visual cortex, a non pain-related region of the brain did not change due to CCI or acetic acid treatments, suggesting that ΔFosB expression to stress and pain presents a regional specificity. The casual relationship between chronic pain and the ΔFosB protein need further exploration.

A number of immunostaining studies show that ΔFosB expresses predominately in projection neurons (Perrotti et al., 2004; Lobo et al., 2013; Nomaru et al., 2014). In the prefrontal cortex, ΔFosB exists in several layers of the cortex, in particular in layers II/III and V. The ΔFosB staining was again localized in the neurons, with no colocalization with GFAP, an astroglial marker seen primarily within cortical pyramidal neurons; over 90% of the ΔFosB+ cells colabeled with vesicular glutamate transporter 1 (vGluT1; a marker of glutamatergic neurons), with little colabeling seen with markers for various GABAergic interneurons (i.e., calbindin, parvalbumin, or calretinin; Perrotti et al., 2004). Based on these data, we predict that chronic stress elevates the ΔFosB protein expression in pyramidal neurons in mPFC.

## Conclusion

Our results clearly show the expression of ΔFosB was significantly elevated in rats undergoing chronic, but not acute, stress-induced pain. The ΔFosB protein expression profile to different stimuli supports the role of ΔFosB in neuronal plasticity and suggests that it could be a useful molecular marker of sustained pain. Further research is needed to improve our understanding of both the upstream mechanism leading to ΔFosB protein production and the downstream mechanism by which the ΔFosB protein might participate in plasticity.

## Author Contributions

Y-MZ and GZ conceived the idea, supervised the research, analyzed the data, and wrote the manuscript. HW conducted the research and wrote the manuscript. All other authors conceived the idea, analyzed the data, and wrote the manuscript. We thank Dr. Diane Baronas-Lowell for proofreading this manuscript.

## Conflict of Interest Statement

The authors declare that the research was conducted in the absence of any commercial or financial relationships that could be construed as a potential conflict of interest.

## References

[B1] Al-Chaer E. D. Kawasaki M. Pasricha P. J. (2000). A new model of chronic visceral hypersensitivity in adult rats induced by colon irritation during postnatal development. *Gastroenterology* 119 1276–1285. 10.1053/gast.2000.19576 11054385

[B2] Amath A. Foster J. A. Sidor M. M. (2012). Developmental alterations in CNS stress-related gene expression following postnatal immune activation. *Neuroscience* 220 90–99. 10.1016/j.neuroscience.2012.06.037 22732504

[B3] Andersson M. Westin J. E. Cenci M. A. (2003). Time course of striatal DeltaFosB-like immunoreactivity and prodynorphin mRNA levels after discontinuation of chronic dopaminomimetic treatment. *Eur. J. Neurosci.* 17 661–666. 10.1046/j.1460-9568.2003.02469.x 12581184

[B4] Baliki M. N. Mansour A. R. Baria A. T. Apkarian A. V. (2014). Functional reorganization of the default mode network across chronic pain conditions. *PLoS ONE* 9:e106133 10.1371/journal.pone.0106133 PMC4152156 25180885

[B5] Bennett G. J. Xie Y. K. (1988). A peripheral mononeuropathy in rat that produces disorders of pain sensation like those seen in man. *Pain* 33 87–107. 10.1016/0304-3959(88)90209-6 2837713

[B6] Bouhassira D. Lanteri-Minet M. Attal N. Laurent B. Touboul C. (2008). Prevalence of chronic pain with neuropathic characteristics in the general population. *Pain* 136 380–387. 10.1016/j.pain.2007.08.013 17888574

[B7] Cash J. L. White G. E. Greaves D. R. (2009). Chapter 17. Zymosan-induced peritonitis as a simple experimental system for the study of inflammation. *Methods Enzymol.* 461 379–396. 10.1016/S0076-6879(09)05417-2 19480928

[B8] Charmandari E. Kino T. Souvatzoglou E. Chrousos G. P. (2003). Pediatric stress: hormonal mediators and human development. *Horm. Res.* 59 161–179. 10.1159/000069325 12649570

[B9] Chen J. Zhang Y. Kelz M. B. Steffen C. Ang E. S. Zeng L. (2000). Induction of cyclin-dependent kinase 5 in the hippocampus by chronic electroconvulsive seizures: role of [Delta]FosB. *J. Neurosci.* 20 8965–8971. 11124971 10.1523/JNEUROSCI.20-24-08965.2000 PMC6773018

[B10] Chen Z. Y. Zhang X. W. Yu L. Hua R. Zhao X. P. Qin X. (2015). Spinal toll-like receptor 4-mediated signalling pathway contributes to visceral hypersensitivity induced by neonatal colonic irritation in rats. *Eur. J. Pain* 19 176–186. 10.1002/ejp.534 24842692

[B11] Chey W. D. Kurlander J. Eswaran S. (2015). Irritable bowel syndrome: a clinical review. *JAMA* 313 949–958. 10.1001/jama.2015.0954 25734736

[B12] Covington H. E. III Lobo M. K. Maze I. Vialou V. Hyman J. M. Zaman S. (2010). Antidepressant effect of optogenetic stimulation of the medial prefrontal cortex. *J. Neurosci.* 30 16082–16090. 10.1523/JNEUROSCI.1731-10.2010 21123555 PMC3004756

[B13] Devoize L. Alvarez P. Monconduit L. Dallel R. (2011). Representation of dynamic mechanical allodynia in the ventral medial prefrontal cortex of trigeminal neuropathic rats. *Eur. J. Pain* 15 676–682. 10.1016/j.ejpain.2010.11.017 21316272

[B14] Garner B. Wood S. J. Pantelis C. Van Den Buuse M. (2007). Early maternal deprivation reduces prepulse inhibition and impairs spatial learning ability in adulthood: no further effect of post-pubertal chronic corticosterone treatment. *Behav. Brain Res.* 176 323–332. 10.1016/j.bbr.2006.10.020 17097157

[B15] Green M. K. Rani C. S. Joshi A. Soto-Pina A. E. Martinez P. A. Frazer A. (2011). Prenatal stress induces long term stress vulnerability, compromising stress response systems in the brain and impairing extinction of conditioned fear after adult stress. *Neuroscience* 192 438–451. 10.1016/j.neuroscience.2011.06.041 21723377

[B16] Guida F. Luongo L. Marmo F. Romano R. Iannotta M. Napolitano F. (2015). Palmitoylethanolamide reduces pain-related behaviors and restores glutamatergic synapses homeostasis in the medial prefrontal cortex of neuropathic mice. *Mol. Brain* 8:47 10.1186/s13041-015-0139-5 PMC4532244 26260027

[B17] Hall F. S. (1998). Social deprivation of neonatal, adolescent, and adult rats has distinct neurochemical and behavioral consequences. *Crit. Rev. Neurobiol.* 12 129–162. 10.1615/CritRevNeurobiol.v12.i1-2.50 9444483

[B18] Herdegen T. Leah J. D. (1998). Inducible and constitutive transcription factors in the mammalian nervous system: control of gene expression by Jun, Fos and Krox, and CREB/ATF proteins. *Brain Res. Brain Res. Rev.* 28 370–490. 10.1016/S0165-0173(98)00018-6 9858769

[B19] Hiroi N. Marek G. J. Brown J. R. Ye H. Saudou F. Vaidya V. A. (1998). Essential role of the fosB gene in molecular, cellular, and behavioral actions of chronic electroconvulsive seizures. *J. Neurosci.* 18 6952–6962. 9712664 10.1523/JNEUROSCI.18-17-06952.1998 PMC6792966

[B20] Hulshof H. J. Novati A. Sgoifo A. Luiten P. G. Den Boer J. A. Meerlo P. (2011). Maternal separation decreases adult hippocampal cell proliferation and impairs cognitive performance but has little effect on stress sensitivity and anxiety in adult Wistar rats. *Behav. Brain Res.* 216 552–560. 10.1016/j.bbr.2010.08.038 20816703

[B21] Ji R. R. Xu Z. Z. Gao Y. J. (2014). Emerging targets in neuroinflammation-driven chronic pain. *Nat. Rev. Drug Discov.* 13 533–548. 10.1038/nrd4334 24948120 PMC4228377

[B22] Jorissen H. J. Ulery P. G. Henry L. Gourneni S. Nestler E. J. Rudenko G. (2007). Dimerization and DNA-binding properties of the transcription factor DeltaFosB. *Biochemistry* 46 8360–8372. 10.1021/bi700494v 17580968

[B23] Kelz M. B. Chen J. Carlezon W. A. Jr. Whisler K. Gilden L. Beckmann A. M. (1999). Expression of the transcription factor deltaFosB in the brain controls sensitivity to cocaine. *Nature* 401 272–276. 10.1038/45790 10499584

[B24] Lehmann M. L. Herkenham M. (2011). Environmental enrichment confers stress resiliency to social defeat through an infralimbic cortex-dependent neuroanatomical pathway. *J. Neurosci.* 31 6159–6173. 10.1523/JNEUROSCI.0577-11.2011 21508240 PMC3094574

[B25] Lobo M. K. Zaman S. Damez-Werno D. M. Koo J. W. Bagot R. C. Dinieri J. A. (2013). DeltaFosB induction in striatal medium spiny neuron subtypes in response to chronic pharmacological, emotional, and optogenetic stimuli. *J. Neurosci.* 33 18381–18395. 10.1523/JNEUROSCI.1875-13.2013 24259563 PMC3834048

[B26] Luis-Delgado O. E. Barrot M. Rodeau J. L. Ulery P. G. Freund-Mercier M. J. Lasbennes F. (2006). The transcription factor DeltaFosB is recruited by inflammatory pain. *J. Neurochem.* 98 1423–1431. 10.1111/j.1471-4159.2006.03970.x 16787404

[B27] Martinez V. Thakur S. Mogil J. S. Tache Y. Mayer E. A. (1999). Differential effects of chemical and mechanical colonic irritation on behavioral pain response to intraperitoneal acetic acid in mice. *Pain* 81 179–186. 10.1016/S0304-3959(99)00008-1 10353506

[B28] Mayer E. A. Collins S. M. (2002). Evolving pathophysiologic models of functional gastrointestinal disorders. *Gastroenterology* 122 2032–2048. 10.1053/gast.2002.33584 12055608

[B29] McClung C. A. Nestler E. J. (2003). Regulation of gene expression and cocaine reward by CREB and DeltaFosB. *Nat. Neurosci.* 6 1208–1215. 10.1038/nn1143 14566342

[B30] McClung C. A. Ulery P. G. Perrotti L. I. Zachariou V. Berton O. Nestler E. J. (2004). DeltaFosB: a molecular switch for long-term adaptation in the brain. *Brain Res. Mol. Brain Res.* 132 146–154. 10.1016/j.molbrainres.2004.05.014 15582154

[B31] Meller S. T. Gebhart G. F. (1997). Intraplantar zymosan as a reliable, quantifiable model of thermal and mechanical hyperalgesia in the rat. *Eur. J. Pain* 1 43–52. 10.1016/S1090-3801(97)90052-5 15102428

[B32] Metz A. E. Yau H. J. Centeno M. V. Apkarian A. V. Martina M. (2009). Morphological and functional reorganization of rat medial prefrontal cortex in neuropathic pain. *Proc. Natl. Acad. Sci. U.S.A.* 106 2423–2428. 10.1073/pnas.0809897106 19171885 PMC2650172

[B33] Morgan J. I. Curran T. (1995). Immediate-early genes: ten years on. *Trends Neurosci.* 18 66–67. 10.1016/0166-2236(95)80022-T 7537412

[B34] Morris T. A. Jafari N. Delorenzo R. J. (2000). Chronic DeltaFosB expression and increased AP-1 transcription factor binding are associated with the long term plasticity changes in epilepsy. *Brain Res. Mol. Brain Res.* 79 138–149. 10.1016/S0169-328X(00)00112-1 10925151

[B35] Nakabeppu Y. Nathans D. (1991). A naturally occurring truncated form of FosB that inhibits Fos/Jun transcriptional activity. *Cell* 64 751–759. 10.1016/0092-8674(91)90504-R 1900040

[B36] Nestler E. J. (2015). FosB: a transcriptional regulator of stress and antidepressant responses. *Euro. J. Pharmacol.* 753 66–72. 10.1016/j.ejphar.2014.10.034 PMC4380559 25446562

[B37] Nestler E. J. Barrot M. Self D. W. (2001). DeltaFosB: a sustained molecular switch for addiction. *Proc. Natl. Acad. Sci. U.S.A.* 98 11042–11046. 10.1073/pnas.191352698 11572966 PMC58680

[B38] Nestler E. J. Kelz M. B. Chen J. (1999). DeltaFosB: a molecular mediator of long-term neural and behavioral plasticity. *Brain Res.* 835 10–17. 10.1016/S0006-8993(98)01191-3 10448191

[B39] Nomaru H. Sakumi K. Katogi A. Ohnishi Y. N. Kajitani K. Tsuchimoto D. (2014). Fosb gene products contribute to excitotoxic microglial activation by regulating the expression of complement C5a receptors in microglia. *Glia* 62 1284–1298. 10.1002/glia.22680 24771617 PMC4090226

[B40] Perrotti L. I. Hadeishi Y. Ulery P. G. Barrot M. Monteggia L. Duman R. S. (2004). Induction of deltaFosB in reward-related brain structures after chronic stress. *J. Neurosci.* 24 10594–10602. 10.1523/JNEUROSCI.2542-04.2004 15564575 PMC6730117

[B41] Rentesi G. Antoniou K. Marselos M. Fotopoulos A. Alboycharali J. Konstandi M. (2010). Long-term consequences of early maternal deprivation in serotonergic activity and HPA function in adult rat. *Neurosci. Lett.* 480 7–11. 10.1016/j.neulet.2010.04.054 20435091

[B42] Rygh L. J. Svendsen F. Hole K. Tjolsen A. (2001). Increased spinal N-methyl-D-aspartate receptor function after 20 h of carrageenan-induced inflammation. *Pain* 93 15–21. 10.1016/S0304-3959(01)00286-X 11406334

[B43] Sheng M. Greenberg M. E. (1990). The regulation and function of c-fos and other immediate early genes in the nervous system. *Neuron* 4 477–485. 10.1016/0896-6273(90)90106-P 1969743

[B44] Toth C. Lander J. Wiebe S. (2009). The prevalence and impact of chronic pain with neuropathic pain symptoms in the general population. *Pain Med.* 10 918–929. 10.1111/j.1526-4637.2009.00655.x 19594844

[B45] Van den Bergh B. R. Van Calster B. Smits T. Van Huffel S. Lagae L. (2008). Antenatal maternal anxiety is related to HPA-axis dysregulation and self-reported depressive symptoms in adolescence: a prospective study on the fetal origins of depressed mood. *Neuropsychopharmacology* 33 536–545. 10.1038/sj.npp.1301540 17507916

[B46] Vialou V. Bagot R. C. Cahill M. E. Ferguson D. Robison A. J. Dietz D. M. (2014). Prefrontal cortical circuit for depression- and anxiety-related behaviors mediated by cholecystokinin: role of DeltaFosB. *J. Neurosci.* 34 3878–3887. 10.1523/JNEUROSCI.1787-13.2014 24623766 PMC3951691

[B47] Vialou V. Robison A. J. Laplant Q. C. Covington H. E. III Dietz D. M. Ohnishi Y. N. (2010). DeltaFosB in brain reward circuits mediates resilience to stress and antidepressant responses. *Nat. Neurosci.* 13 745–752. 10.1038/nn.2551 20473292 PMC2895556

[B48] Wang Q. Shao F. Wang W. (2015). Maternal separation produces alterations of forebrain brain-derived neurotrophic factor expression in differently aged rats. *Front. Mol. Neurosci.* 8:49 10.3389/fnmol.2015.00049 PMC4555027 26388728

[B49] Werme M. Messer C. Olson L. Gilden L. Thoren P. Nestler E. J. (2002). Delta FosB regulates wheel running. *J. Neurosci.* 22 8133–8138. 12223567 10.1523/JNEUROSCI.22-18-08133.2002 PMC6758121

[B50] Woolf C. J. Costigan M. (1999). Transcriptional and posttranslational plasticity and the generation of inflammatory pain. *Proc. Natl. Acad. Sci. U.S.A.* 96 7723–7730. 10.1073/pnas.96.14.7723 10393888 PMC33609

[B51] Wouters M. M. Van Wanrooy S. Casteels C. Nemethova A. De Vries A. Van Oudenhove L. (2012). Altered brain activation to colorectal distention in visceral hypersensitive maternal-separated rats. *Neurogastroenterol. Motil.* 24:e297 10.1111/j.1365-2982.2012.01919.x 22509925

[B52] Zachariou V. Bolanos C. A. Selley D. E. Theobald D. Cassidy M. P. Kelz M. B. (2006). An essential role for DeltaFosB in the nucleus accumbens in morphine action. *Nat. Neurosci.* 9 205–211. 10.1038/nn1636 16415864

[B53] Zhang G. Zhao B. X. Hua R. Kang J. Shao B. M. Carbonaro T. M. (2015). Hippocampal microglial activation and glucocorticoid receptor down-regulation precipitate visceral hypersensitivity induced by colorectal distension in rats. *Neuropharmacology* 102 295–303. 10.1016/j.neuropharm.2015.11.028 26656865

[B54] Zhang Y. Crofton E. J. Li D. Lobo M. K. Fan X. Nestler E. J. (2014). Overexpression of DeltaFosB in nucleus accumbens mimics the protective addiction phenotype, but not the protective depression phenotype of environmental enrichment. *Front. Behav. Neurosci.* 8:297 10.3389/fnbeh.2014.00297 PMC4148937 25221490

[B55] Zhou Q. Fillingim R. B. Riley J. L. III Malarkey W. B. Verne G. N. (2010). Central and peripheral hypersensitivity in the irritable bowel syndrome. *Pain* 148 454–461. 10.1016/j.pain.2009.12.005 20074857 PMC2913434

